# Orientation microscopy study of cryogenic-temperature friction stir processed AA6xxx sheet: microstructure and second phase particles evolution

**DOI:** 10.1186/s42649-026-00133-x

**Published:** 2026-06-10

**Authors:** Aman Gupta, Khushahal Thool, Shi-Hoon Choi

**Affiliations:** Department of Advanced Components and Materials Engineering, Sunchon National University, Sunchon-Si , 57922 Republic of Korea

**Keywords:** FSP, Cryogenic cooling, Microhardness, Intermetallics, EBSD, Deep learning

## Abstract

Cryogenic-temperature friction stir processing (CT-FSP) was performed on AA6xxx sheets at 600 RPM and 500 mm/min using in-process liquid N_2_ cooling to investigate microstructure, precipitate evolution, and the resulting mechanical response. Multiscale characterization EBSD, SEM/EDS and microhardness tests were conducted. CT-FSP produced a highly refined predominantly equiaxed stir zone (SZ) with average grain sizes of approximately 1.6–1.8 µm at the surface and mid-thickness, and slightly coarser grains (~ 4.4 µm) near the bottom and within the thermomechanically affected zone (TMAZ). Dynamic recrystallization, evidenced by a high fraction of high-angle grain boundaries and low KAM, dominates the SZ. The recrystallization fraction varies laterally and through the thickness due to heterogeneous strain and thermal fields. Second phase particles experience mechanical fragmentation, partial solvus dissolution, and rapid reprecipitation during CT-FSP, yielding a finer and more uniformly dispersed distribution in the upper SZ, while coarser Al–Fe–Si and Mg_2_Si particles persist near the bottom/TMAZ. Deep-learning-based image segmentation reveals that the mean equivalent-circle diameters of Al–Fe–Si and Mg_2_Si phases decrease from ~ 0.97 µm and 0.80 µm in the as-received sheet to ~ 0.6–0.8 µm locally after CT-FSP, accompanied by reduced nearest-neighbor spacing in the SZ. This localized particles refinement and redistribution, together with extensive DRX, accounts for the combined SZ softening and TMAZ hardening, establishing a clear microstructure-property linkage in cryogenic FSPed AA6xxx sheet.

## Introduction

Friction stir processing (FSP) is a solid-state surface modification technique derived from friction stir welding, in which a rotating, non-consumable tool induces intense local plastic deformation and stirring to generate refined microstructures (Gupta et al. 2025a). Through severe plastic shear and controlled thermal input, FSP promotes subgrain formation, dynamic recovery (DRV), and dynamic recrystallization (DRX), enabling grain sizes to be reduced from tens of micrometers to the submicron or even nanometer scale without melting (Gupta et al. 2025b). The technique has been widely applied to eliminate casting defects, fragment coarse intermetallic phases, and produce homogeneously refined stir zones (SZs) that enhance local ductility and formability (Silvestri et al. 2024). Coupled approaches such as multi-pass FSP (Rubtsov et al. 2023; Najar et al. 2025), modification in tool geometry (Pouraliakbar et al. 2024; Zhang et al. 2025), and the incorporation of reinforcing phases (Ravi et al. 2025; Moustafa et al. 2024) further explore strain and diffusional-driven phenomena to control precipitate fragmentation, particle distribution, and grain boundary character, ultimately optimizing mechanical performance (Pouraliakbar et al. 2024, 2022). Li et al. (2025) examined the effect of FSP on as-cast and hot rolled (HR-FSP) aluminium matrix composites processed at various tool rotation speeds (800, 1000, and 1200 RPM). Although the average grain size (GS_Avg_) decreased with increasing rotational speed in both cases, HR-FSP consistently produced finer grains. Orientation microscopy indicated that recrystallization was primarily governed by discontinuous DRX (DDRX), with additional contribution from continuous DRX (CDRX). The pre-existing dislocation substructure from hot rolling accelerated recrystallization during FSP, yielding superior mechanical properties particularly at 1200 rpm, where HR-FSP achieved an average hardness of 59.78 Hv and enhanced strength–ductility balance compared with the as-cast FSPed counterpart (Li et al. 2025). Recently, considerable interest has focused on reducing the thermal cycle by employing alternative cooling media during processing (Wang et al. 2024). Shortening the thermal cycle significantly influences microstructural and precipitate evolution in FSPed aluminum alloys. Consequently, the concept of “cryogenic FSP” has emerged and has been explored in recent studies involving both steels (Cao et al. 2023) and aluminium alloys (Khorrami et al. 2017; Kumar et al. 2021).

Cryogenic-temperature FSP (CT-FSP) extends conventional processing by applying active cooling typically liquid nitrogen during tool rotation, thereby markedly reducing the peak and dwell temperatures experienced by the material (Huang 2016). The significantly lowered thermal budget suppresses recovery and post-deformation grain growth, stabilizing the ultrafine grains formed by DRX and increasing the fraction of high-angle grain boundaries (HAGBs) (Gupta et al. 2025a). CT-FSP also alters solute and second-phase particle behavior, reduced temperatures limit extensive solvus dissolution but heighten the roles of mechanical fragmentation and rapid, localized reprecipitation, resulting in a finer and more homogeneous dispersion of Mg- and Fe-bearing precipitates in Al–Mg–Si alloys (Gupta et al. 2025a). Despite persistent through-thickness and lateral gradients in cooling and strain, CT-FSP microstructures reflect a balance between mechanical stirring, suppressed diffusion, and heterogeneous heat extraction. In a representative study on AA7075–SiC composites (Kumar et al. 2021), CT-FSP at 1025 RPM produced a highly refined and uniformly recrystallized nugget. The cast dendritic structure and porosity were eliminated, and the average matrix grain size was reduced to ~ 1–3 µm. EBSD analysis revealed signatures of CDRX, random boundary misorientation distributions, and a high HAGB fraction. Nanoscale SiC reinforcements (3 wt.%) became well redistributed, often decorating grain boundaries and, when uniformly dispersed, promoted grain refinement through Zener pinning and particle-stimulated nucleation. Conversely, severe SiC agglomeration (at 5 wt.% addition) resulted in local microstructural inhomogeneity and coarser regions (Kumar et al. 2021). Our previous investigations on room-temperature FSP (RT-FSP) of AA6xxx showed a heterogeneous grain-size distribution when using a rotational speed of 800 RPM (Gupta et al. 2025b). Pronounced refinement to ~ 2 µm occurred in the SZ, which included the surface and mid-thickness regions, whereas the bottom region exhibited a much coarser grain structure (~ 14 µm) accompanied by strong orientation gradients characteristic of a thermomechanically affected zone (TMAZ) (Gupta et al. 2025b). In subsequent work, CT-FSP at the same rotational speed (800 RPM) on AA6xxx sheet produced considerably more homogeneous refinement across the SZ (Gupta et al. 2025a). Grain sizes in the surface, center, and bottom regions were reduced to ~ 2–4 µm, compared with ~ 18 µm in the as-received AA6xxx sheet. A direct comparison of RT- and CT-FSP at identical RPM revealed that the extent and morphology of the refined regions differ substantially between the two conditions.

Mechanically, CT-FSP produces distinct outcomes compared with RT-FSP. Conventional FSP or RT-FSP generally promotes DRX and grain refinement but may also allow significant recovery and precipitate coarsening due to higher thermal input. Depending on precipitate stability and the presence of reinforcing particles, hardness may be retained or even increased. In contrast, CT-FSP typically yields finer, thermally stabilized grains and modified precipitate states that can either enhance strength through retained nanoscale dispersions or particle pinning or reduce local hardness when precipitation-strengthening is diminished. For example, in CT-FSPed AA7075–SiC composites (Kumar et al. 2021), Vickers hardness in the nugget zone increased from 69 Hv to 106 Hv, and tensile properties improved substantially, with yield and ultimate tensile strengths reaching 391 and 552 MPa, respectively, for the AA7075–3 wt.% SiC composite. These improvements were attributed to Hall–Petch strengthening, Orowan/precipitation strengthening associated with refined precipitates, and the uniform redistribution of SiC particles (Kumar et al. 2021). Importantly, CT-FSP often enhances formability, and delays crack initiation by fragmenting coarse intermetallics and reducing microstructural stress concentrators. Reported trends in AA6xxx alloys show SZ softening resulting from partial precipitate dissolution, accompanied by improved bendability and hemmability effects linked to homogeneous DRX, reduced strain localization, and beneficial texture evolution under cryogenic cooling (Gupta et al. 2025a). Improving mechanical properties remains a central goal of FSP. As reported in Gupta et al. 2025b, the initial microhardness of the AA6xxx base material was 87 ± 6 Hv. After RT-FSP, the SZ softened to 67–86 Hv, while the TMAZ hardened to 92–98 Hv. These contrasts reflect zone-specific differences in precipitate state, dislocation density, and subgrain evolution under RT versus CT conditions. Notably, SZ softening is not universal as in our work on AA5083 (Alluri et al. 2025), the SZ hardness increased relative to the base metal. For AA5083 processed at 800 RPM, the highest hardness occurred near the surface (94.7 Hv), decreasing modestly through the thickness (91.1 Hv at mid-thickness and 89.1 Hv at the bottom). Overall, the processed region exhibited a hardness range of ~ 76–96 Hv, compared with 87–88.6 Hv for the base material, indicating surface-biased strengthening of the SZ (Alluri et al. 2025).

Motivated by these coupled microstructural–mechanical effects and by earlier CT-FSP studies conducted at higher rotation speeds (Khorrami et al. 2017; Huang 2016; Satyanarayana and Kumar 2021), the present work investigates AA6xxx sheet processed at 600 RPM (500 mm/min) with in-process liquid-nitrogen cooling. The objective is to clarify how reduced rotation speed and cryogenic heat extraction jointly influence grain refinement, DRX mechanisms, precipitate evolution, and resulting hardness. Although extensive work exists on FSP of AA6xxx (Moustafa et al. 2024; Cai et al. 2025; Nabi et al. 2025), little attention has been given to CT-FSP at comparatively low tool speeds. Using multiscale characterization EBSD for grain size and dislocation metrics (kernel average misorientation, KAM), SEM/EDS for second-phase particle assessment, and microhardness mapping, we aim to (1) quantify through-thickness grain size and DRX variations at reduced rotational speed; (2) track transformation, fragmentation, and reprecipitation of Mg_2_Si and Al–Fe–Si phases; and (3) correlate microstructural changes with hardness distributions to guide CT-FSP parameter selection for improved automotive panel performance.

## Experimental

**Fig. 1:**
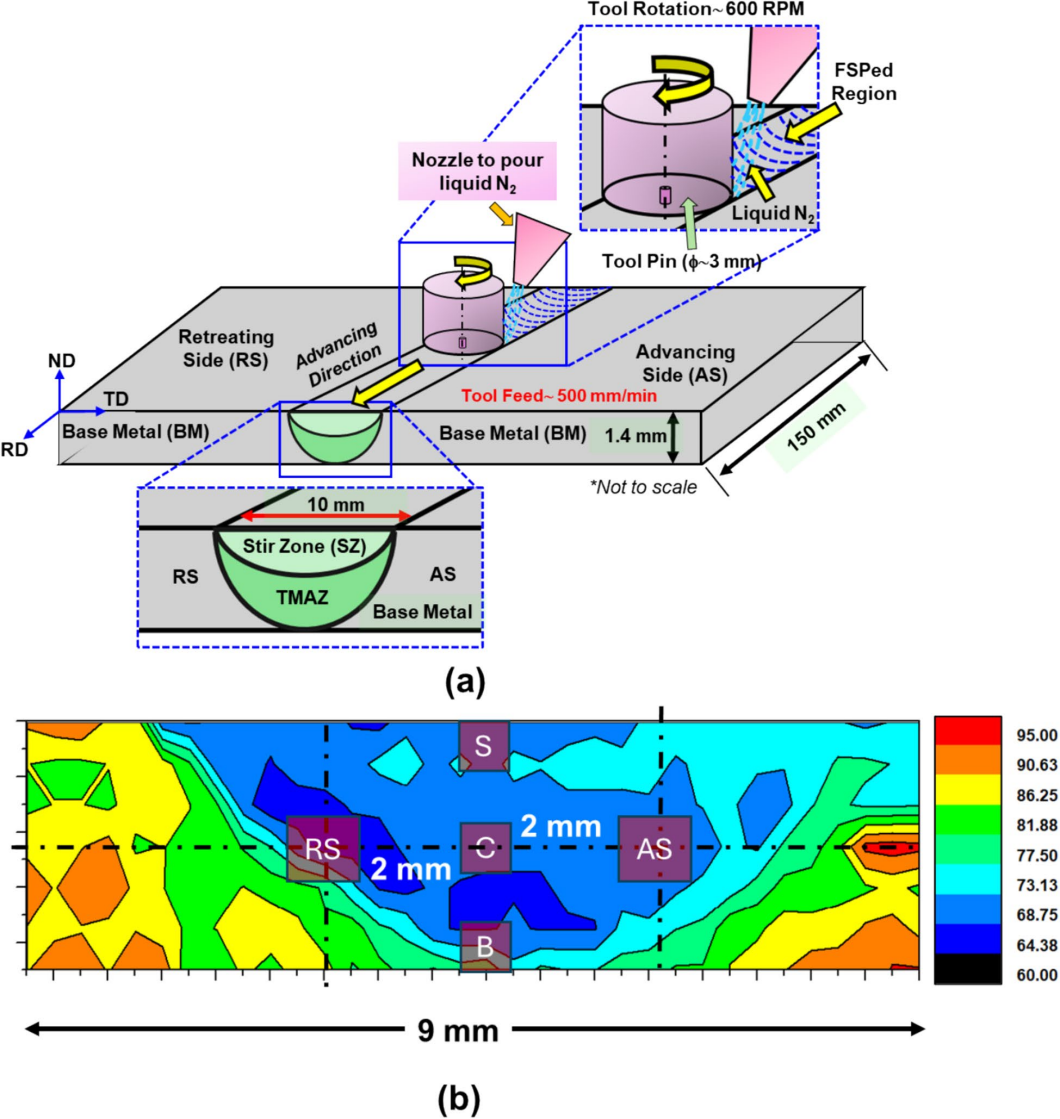
**a** Schematic of the cryogenic-temperature friction stir processing (CT-FSP) setup used in this study. **b** Microhardness contour map for the 600-RPM CT-FSPed sample. Representative locations of SEM and EBSD measurements are indicated on the contour map. Note: S = surface, C = center, B = bottom, AS = advancing side, RS = retreating side. Liquid nitrogen was delivered behind the tool to achieve rapid in-process cooling

AA6xxx rolled sheets (1.4 mm thickness), originally strip-cast to 80% reduction and subsequently solution-annealed at 540 °C for 30 min, were used as the starting material. The chemical composition (wt.%) of the AA6xxx sheet was: Si 1.21, Mg 0.67, Fe 0.16, Mn 0.11, Cu 0.17, with minor additions of Zn, Cr, V, and Ti. Cryogenic friction stir processing (CT-FSP) was conducted using a heat-treated SKD61 steel tool with a 10 mm shoulder diameter and a 3 mm pin diameter (pin length 1 mm). Processing was performed along the rolling direction (tool travel//RD) at a rotational speed of 600 RPM and a traverse speed of 500 mm min^-1^. Liquid nitrogen (approximately − 50 °C working temperature) was continuously dispensed from a rear-mounted nozzle to provide active low-temperature cooling during processing. The CT-FSP pass produced a processed band approximately 10 mm wide and reduced the local sheet thickness within the stirred region to ~ 1.2 mm. For simplicity, the unprocessed sheet and processed specimens are referred to as “as-received” and “CT-FSP,” respectively. Figure 1a schematically illustrates the CT-FSP configuration. During processing, liquid nitrogen cooling maintained a low temperature environment around the stir zone (SZ), suppressing peak temperatures and enabling rapid heat extraction. The advancing side (AS) and retreating side (RS) are identified relative to tool rotation and travel direction. As the tool advanced, severe plastic deformation combined with spatially non-uniform cryogenic cooling generated heterogeneous thermal and strain gradients within the SZ and the adjoining thermo-mechanically affected zone (TMAZ).

Small samples measuring 12 mm (TD) × 6 mm (ND) were sectioned using a Struers Accutom-50 precision cutter for metallographic preparation. The samples were ground using SiC papers up to 2400 grit and then electropolished for EBSD using a ethanol: perchloric acid (9:1) solution at 24 V for 20 s. EBSD scans were acquired on a field-emission SEM (JEOL JSM-7100F) using a step size of 0.3 µm over 100 × 100 µm^2^ areas at selected positions throughout the processed region (surface, center, bottom, AS, and RS), as indicated in the hardness map in Fig. 1b. Figure 1b presents the microhardness contour for the 600-RPM CT-FSPed sample, revealing distinct hardness variations across the AS and RS. These gradients reflect through-thickness microstructural heterogeneity, which is discussed in the subsequent sections. The surface (S), center (C), and bottom (B) positions marked on the contour correspond to the approximate locations selected for EBSD and SEM analyses.

EBSD post-processing was performed using TSL OIM v7. Grains with a confidence index (CI) < 0.10 were removed, recrystallized grains were identified using a grain orientation spread (GOS) threshold of ≤ 1.5°, and kernel average misorientation (KAM) values were calculated using the 3rd nearest neighbor. SEM imaging (secondary electron mode) and EDS mapping/point analysis were used to characterize second phase particles morphology and chemistry before and after CT-FSP. Microhardness mapping of the processed band was performed using Vickers indents spaced 200 µm apart (approximately 300 indents total) to construct a detailed hardness contour. The measurements were conducted using a CLIMEX MMT-X7 tester with a 100 g load and a 10 s dwell time.

## Results and discussions

### Microstructure and microhardness of FSPed sample

**Fig. 2:**
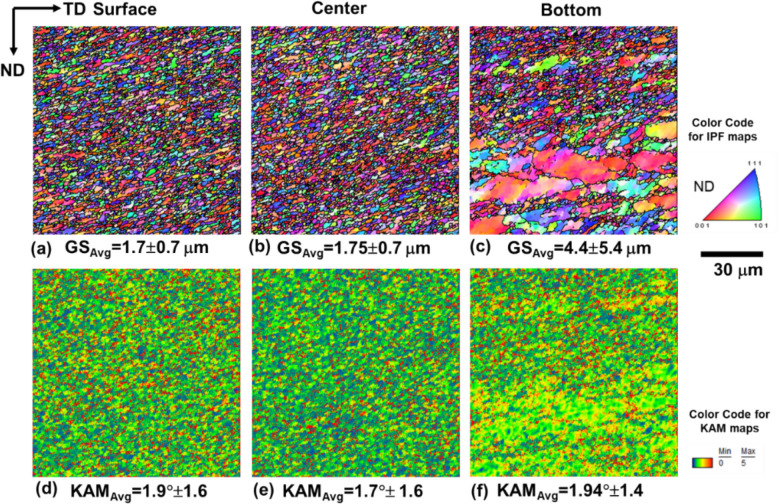
Through-thickness microstructural evolution in the FSPed region. **a**-**c** ND-IPF (inverse pole figure) maps and **d**-**f** Kernel average misorientation (KAM) maps for the surface, center, and bottom regions of the 600 RPM CT-FSPed sample. Note: Average grain size (GS_Avg_) was calculated considering 5° grain tolerance angle. KAM was calculated using the 3rd nearest neighbor. The surface and center correspond to the SZ, whereas the bottom lies near the SZ and TMAZ interface

The as-received (T4) AA6xxx sheet exhibits a coarse, fully recrystallized microstructure with an average grain size of 18 ± 7 μm, a high fraction of HAGBs (~ 93%), and a low kernel average misorientation (KAM) of ~ 0.6°. Details of the initial microstructure have been reported elsewhere (Gupta et al. 2025b) and hence not discussed here. Orientation microscopy was conducted at various positions across the processed zone, as indicated in Fig. 1b. Because temperature and strain fields vary significantly during FSP, microstructural distribution within the SZ and adjacent regions is inherently heterogeneous. Regions in direct contact with the tool pin experience the highest temperatures and the most intense deformation. Figure 2 shows the through-thickness microstructural evolution of the 600-RPM CT-FSPed AA6xxx sample using inverse pole figure (ND-IPF) maps (Fig. 2a–c) and corresponding KAM maps (Fig. 2d–f) at the surface, center, and bottom of the processed zone. At the surface (Fig. 2a, d), intense grain refinement is observed due to high strain rates and direct contact with the rotating shoulder. The average grain size (GS_Avg_) is 1.7 ± 0.7 µm, while the average KAM (1.9° ± 1.6°) indicates moderate local misorientation, consistent with extensive DRX and reduced dislocation density. Although the KAM is higher than in the as-received material, the dominance of HAGBs confirms that recrystallization is largely complete, which can contribute to localized softening. In the center region (Fig. 2b, e), the microstructure remains equiaxed and uniformly refined, with GS_Avg_ ≈ 1.75 µm and KAM_Avg_ ≈ 1.7°. Compared with the surface, the slightly lower KAM values suggest a modestly reduced dislocation content, reflecting a more balanced temperature–strain environment. The fine, homogeneous grain morphology indicates a stable DRX process at the core of the SZ, consistent with previous observations in CT-FSPed aluminum alloys (Gupta et al. 2025a). At the bottom of the processed zone (Fig. 2c, f), a noticeably coarser grain structure (GS_Avg_ ≈ 4.4 ± 5.4 µm) with KAM_Avg_ ≈ 1.94° were observed. This region corresponds to the TMAZ, where lower strain levels and reduced cooling rates hinder complete DRX. The resulting partially recovered microstructure reflects the limited deformation and thermal exposure experienced near the bottom surface.

Interestingly, fine recrystallized grains were observed along the boundaries of the coarser grains in the bottom region. The higher orientation gradients within the coarse grains confirm that this region belongs to the TMAZ. The microstructural transition from surface to bottom highlights the through-thickness heterogeneity typical of FSPed sheets, arising from variations in strain intensity and local thermal exposure. Overall, these results demonstrate that cryogenic cooling during 600-RPM FSP effectively promotes the formation of fine, equiaxed grains throughout the SZ (surface and center), while moderately coarser grains persist at the bottom due to limited deformation and reduced cooling efficiency. Compared with the previously reported 800-RPM CT-FSP condition, the lower rotational speed in the present study results in reduced heat generation, producing a more refined and stable microstructure but also leading to slightly higher KAM values, likely due to increased strain accumulation (Alluri et al. 2025). Alluri et al. (2025) similarly reported higher accumulated strain values (KAM values) at lower rotational speed (800 RPM) compared with 1200 RPM for AA5083 FSPed samples. In the case of the 800 RPM CT-FSP study (Gupta et al. 2025a), the surface-to-bottom region exhibited a relatively homogeneous grain size distribution (GS_Avg_ ≈ 3 ± 1 µm), and KAM values across the thickness were ~ 1°. In contrast, the 600-RPM condition examined here shows more pronounced microstructural heterogeneity. Pouraliakbar et al. (2022) investigated the influence of cooling environment on FSPed Al–Cu–Mg alloys and showed that, under air-cooling, increasing the rotation speed increases the crystallite size during RT-FSP. However, in water-cooled conditions, crystallite size did not vary significantly with rotation speed (Pouraliakbar et al. 2022). Their experimental setup immersed the tool in water, which differs substantially from the present liquid-nitrogen cooling configuration. Consequently, differences in grain size and grain boundary fraction between the 600-RPM and 800-RPM CT-FSPed samples (Gupta et al. 2025a) can be attributed to the combined influence of strain, heat input, and the specific cooling strategy employed.

**Fig. 3:**
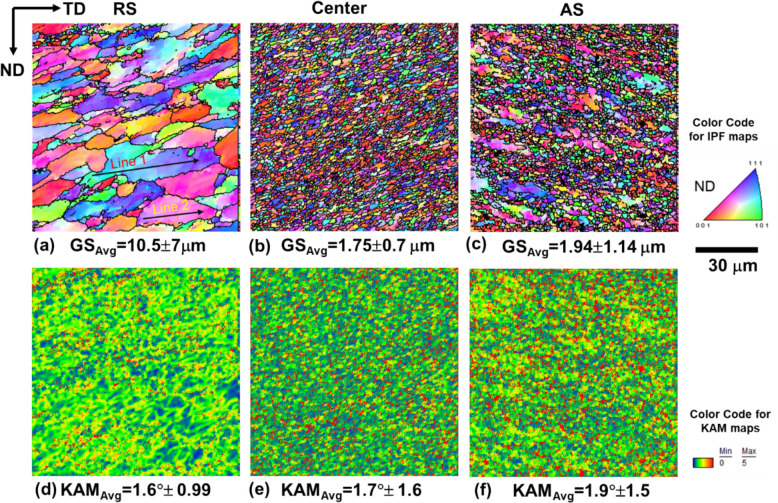
Microstructural evolution along the center line (horizontal direction) in the FSPed region: **a**-**c** ND-IPF maps and **d**-**f** KAM maps for the RS, center, and AS regions of the 600 RPM CT-FSPed sample. Note: Average grain size (GS_Avg_) was calculated considering 5° grain tolerance angle. KAM was calculated using the 3rd nearest neighbor. RS corresponds to the TMAZ, the center represents the SZ and AS denotes the interface between the SZ and TMAZ

Figure 3 illustrates the microstructural evolution across the CT-FSPed AA6xxx sheet at 600 RPM, highlighting the RS, center of the SZ, and AS. ND-IPF maps in Fig. 3a–c reveal grain morphology and distribution, while the corresponding KAM maps in Figs. 3d–f show local misorientation and dislocation density. At the RS (Fig. 3a, d), elongated or slightly distorted grains appear near the SZ–TMAZ boundary, with an GS_Avg_ of 10.5 ± 7 μm. The KAM values are relatively low (up to 1.6°), indicating limited deformation and incomplete DRX. Several coarse grains retain pronounced orientation gradients, as indicated by lines 1 and 2, which show cumulative misorientations of 22° and 16°, respectively. The lower strain rate at the RS arising from the opposite direction of material flow relative to the tool rotation promotes moderate grain refinement and leaves behind a residual substructure. Irregular grain boundaries observed in this region reflect partial boundary migration during FSP. At the center of the SZ (Fig. 3b, e), a uniformly refined, equiaxed grain structure is observed. Grain size is reduced to 1.75 ± 0.7 μm, and KAM_Avg_ ≈ 1.7°, indicating a dislocation density similar to that in the RS and AS. The equiaxed morphology and high HAGB fraction confirm a fully recrystallized microstructure. Rapid cryogenic cooling suppresses grain coarsening and stabilizes the submicron grain structure, consistent with prior studies on cryogenically processed aluminum alloys (Gupta et al. 2025a). At the AS (Fig. 3c, f), grain morphology remains equiaxed and relatively fine (GS_Avg_ ≈ 1.94 ± 1.14 μm). The slightly elevated KAM_Avg_ (~ 1.9°) suggests localized strain accumulation and dislocation entanglement. The asymmetry between the AS and RS arises from variations in material flow and thermal gradients, as the AS encounters higher strain rates and greater frictional heating. Alluri et al. (2025) reported similar behavior in AA5083 FSPed at 800 RPM, where finite element simulations revealed higher peak temperatures and larger effective strains on the AS than on the RS. These differences in thermo-mechanical conditions directly influence the resulting microstructure and the distribution of second-phase particles. Consequently, both DRV and CDRX operate simultaneously at the AS, producing a transitional microstructure between the fully recrystallized SZ and the partially deformed TMAZ (Alluri et al. 2025). Overall, the IPF and KAM analyses confirm that CT-FSP at 600 RPM produces a refined and thermally stable SZ, with a gradual increase in dislocation density from the center (lowest) toward the AS (highest). The observed microstructural heterogeneity reflects the interplay among strain, temperature, and cooling rate, demonstrating controlled DRX under cryogenic conditions. In comparison with higher-speed CT-FSP (800 RPM), the present 600-RPM condition exhibits a more heterogeneous grain-size distribution. Although regional selection can influence quantitative values, both tool rotation speed and cooling rate play critical roles in determining the final microstructure (Zhao et al. 2019).

**Fig. 4:**
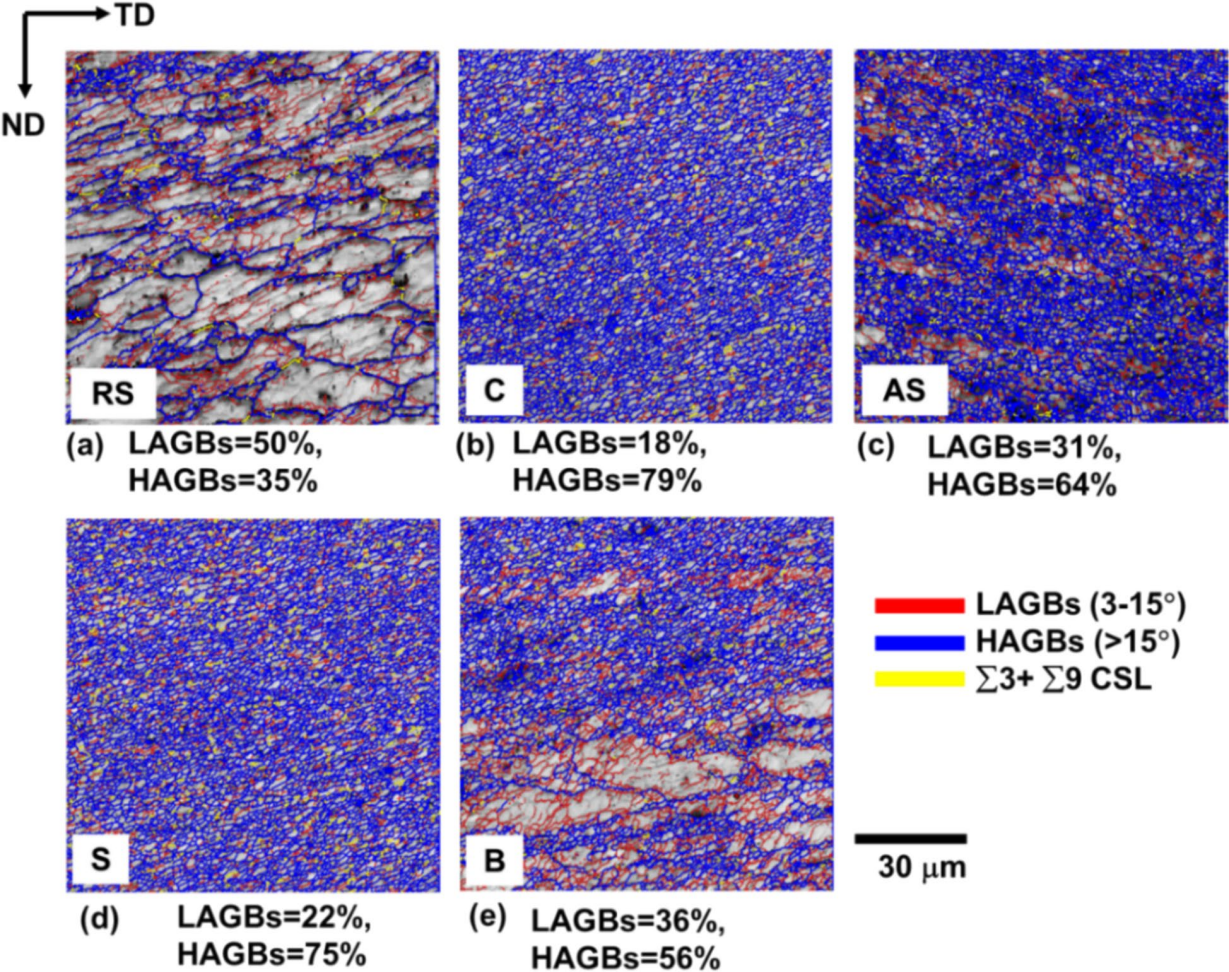
Image quality (IQ) map superimposed with grain boundaries for **a** RS, **b** center, **c** AS, **d** surface, and **e** bottom regions. Red boundaries indicate LAGBs (3–15°), and blue boundaries indicate HAGBs (> 15°). The scale bar is common to all images. Bright regions correspond to high IQ values, whereas darker regions including grain boundaries and particle sites exhibit lower IQ

Figure 4 complements Figs. 2 and 3 by showing IQ + GB maps of the same regions (RS, center, AS, surface, and bottom), allowing visualization of grain boundary characteristics and EBSD indexing quality. Figure 4a–c correspond to the RS, center, and AS regions, respectively. At the RS (Fig. 4a), IQ contrast is slightly reduced near grain boundaries, indicating local strain-induced lattice distortion, while coarse grains exhibit high IQ values. Grain boundary analysis reveals ~ 35% HAGBs and ~ 50% LAGBs, with occasional CSL boundaries (Σ3 + Σ9). The coexistence of LAGBs and HAGBs is characteristic of partially recovered microstructures, confirming moderate DRX accompanied by DRV. The presence of elongated grains along the shear direction suggests that strain accommodation occurs primarily through dislocation glide rather than grain rotation alone. Coincidence site lattice (CSL) boundaries represent special grain boundaries characterized by a high degree of atomic coincidence (low Σ values). Although these boundaries generally exhibit lower interfacial energy and greater thermal stability than random high-angle grain boundaries (HAGBs), they can still influence recrystallization by serving as effective sinks for dislocations and by promoting localized stored-energy gradients in their vicinity. Despite their intrinsically low energy and stability, CSL boundaries can facilitate recrystallization because they enable easier dislocation rearrangement and accelerate boundary migration (Tschopp et al. 2012; Zhao et al. 2018). In studies on Al–Mg–Si alloys, CSL analysis has been used to evaluate how FSP and heat input modify boundary character. Low-Σ boundaries such as Σ3 initially increase after processing but decrease at higher heat input or plastic strain, while high-Σ fractions tend to rise (Pouraliakbar et al. 2025). In the present work, CSL (Σ3 + Σ9) boundaries are slightly more prevalent in the SZ (3–4%) than in the TMAZ (< 2%). In the center region (Fig. 4b), the IQ map exhibits bright contrast with sharply defined boundaries, indicating well-indexed and largely strain-free grains. Quantitative analysis reveals a HAGB fraction of ~ 79%, confirming extensive DRX accompanied by substantial subgrain rotation. The relatively low LAGB fraction (~ 18%), combined with the uniform IQ intensity, suggests that the center of the SZ experienced optimal strain–heat synergy, enabling full DRX and facilitating precipitate dissolution. On the AS (Fig. 4c), the IQ contrast decreases slightly, and grain boundaries appear more irregular. The HAGB fraction drops to ~ 64%, whereas the LAGB fraction increases to ~ 31%. This trend is consistent with the microstructural gradient observed in Fig. 3, where partial DRX occurs at the AS–TMAZ transition due to asymmetric strain and thermal gradients. The surface region (Fig. 4d) shows grain boundary characteristics similar to those in the center. A high fraction of HAGBs (~ 75%) and a lower LAGB fraction (~ 22%) confirm that the surface undergoes substantial DRX, aided by direct tool–shoulder contact and rapid cryogenic cooling. The bottom region (Fig. 4e) contains a mixture of refined grains and coarser structures, reflecting incomplete DRX. Here, the HAGB fraction decreases to ~ 56%, while the LAGB fraction rises to ~ 36%. These observations correlate with the lower strain and reduced cooling efficiency near the sheet bottom, which limit recrystallization and promote partial recovery. Collectively, the IQ + GB maps demonstrate that cryogenic FSP at 600 RPM promotes the formation of a refined and thermally stable microstructure dominated by HAGBs across the SZ, while preserving clear strain gradients from the center toward the RS and bottom regions. The suppression of excessive thermal exposure under cryogenic cooling minimizes grain coarsening and enhances the retention of deformation-induced boundaries, particularly in regions subjected to lower strain.

**Fig. 5:**
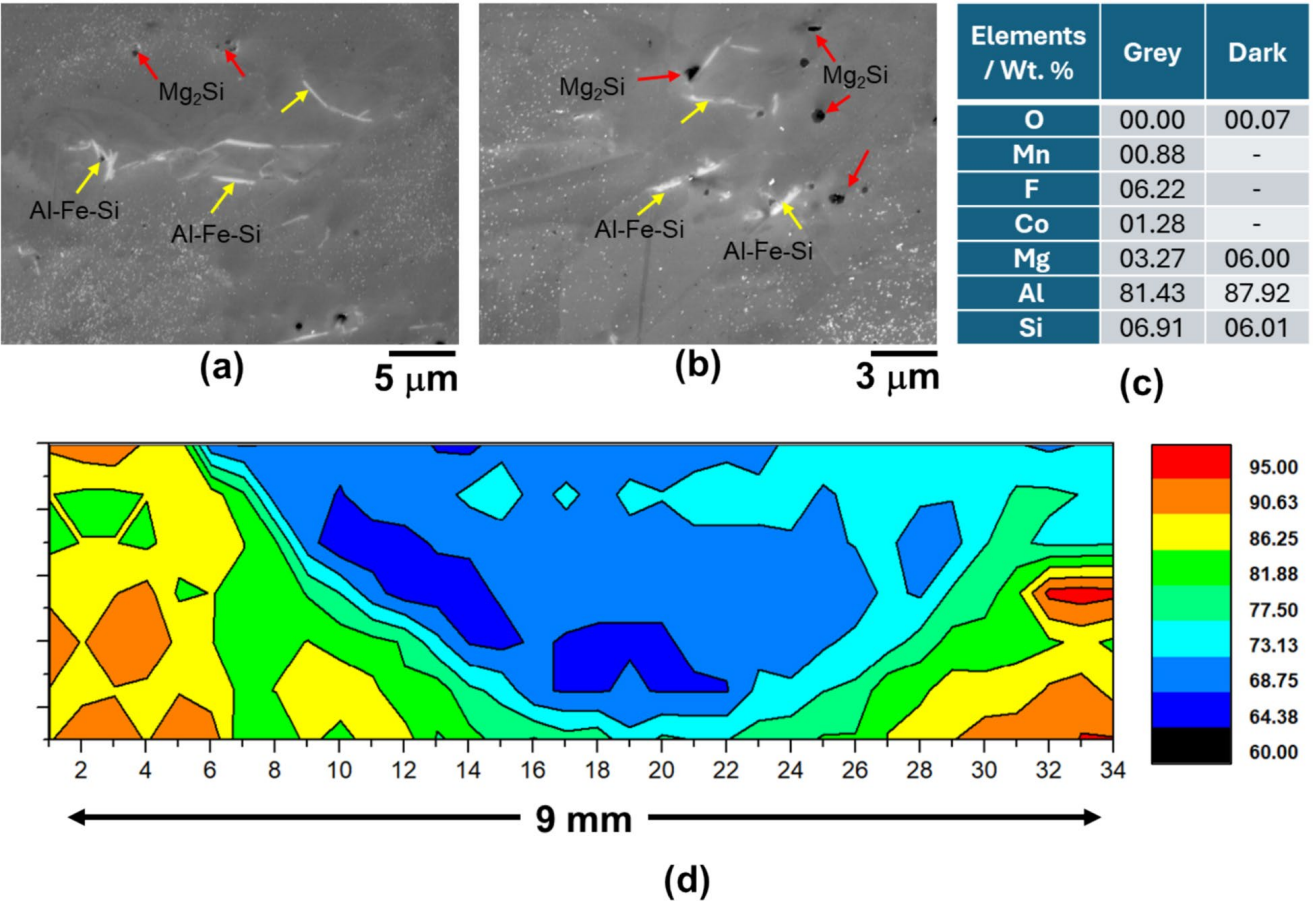
**a**, **b** Scanning electron micrographs (SEM) showing the distribution of second phase particles in the as-received AA6xxx sheet. Two distinct contrasts, grey and dark correspond to different particle types. **c** Elemental distributions obtained from EDS point analysis for grey (Al–Fe–Si) and dark (Mg_2_Si) particles in the as-received sheet. **d** Microhardness contour map for the CT-FSPed sample. Shades of red to orange represent higher hardness values (90–97 Hv), whereas shades of blue denote lower hardness regions (63–75 Hv)

Figure 5 shows the morphology, distribution, and elemental composition of second phase particles present in the as-received AA6xxx sheet prior to CT-FSP. Figure 5a, b display SEM images revealing two distinct particles contrasts: dark and grey. The darker, near-spherical particles correspond to Mg_2_Si, while the grey, elongated or irregularly shaped particles are Al–Fe–Si intermetallics. These precipitates are heterogeneously distributed along the RD, frequently segregated at grain boundaries or within deformed grains (Davidkov et al. 2011; Kumar et al. 2018). The arrows identifying Al–Fe–Si and Mg_2_Si particles are colored yellow and red, respectively. Figure 5c presents EDS point analysis confirming the composition of the two particle types: Mg_2_Si shows elevated Mg and Si peaks, whereas Al–Fe–Si phases exhibit enrichment in Fe and Si, with minor amounts of Mg, Co, and Mn. Image-based quantification using ImageJ indicates that the average particle size of Al–Fe–Si intermetallics is approximately 4 µm, while Mg_2_Si particles range from 0.8 to 1 µm. Overall, the as-received AA6xxx sheet contains a heterogeneous distribution of micro-sized Al–Fe–Si and Mg_2_Si particles, which subsequently influence the deformation and precipitation behavior during FSP.

Figure 5d presents the microhardness contour map of the CT-FSPed region. This map is essential for correlating microstructural features with the observed softening and hardening behavior. Color variations reflect local hardness differences, lower microhardness regions appear in blue, while higher hardness areas appear in yellow, orange, and red. Softening refers to reduced hardness relative to the base material, whereas hardening denotes an increase in microhardness. Regions exhibiting higher hardness (yellow–orange–red) correspond to areas containing coarse grain with strong orientation gradient and subgrain evolution. Conversely, softer regions (blue) are associated with DRX as well as partial dissolution of second phase particles. In the present CT-FSPed sample, the SZ exhibits noticeable softening, with microhardness values between 64 and 73 Hv. In contrast, the TMAZ, shown by green contours, displays moderately increased hardness (77–83 Hv). Moving farther from the SZ–TMAZ interface (left region), a clear hardening trend emerges, as indicated by the yellow and orange contours, corresponding to hardness values of 86–93 Hv. Microhardness of the as-received AA6xxx sample was measured to be 87 ± 6 Hv. Further information about the microhardness transition between the SZ-TMAZ-BM (base metal) is discussed in Sect. “Effect of second phase particles on microhardness”. Softening within the SZ primarily results from precipitate dissolution and dynamic softening, both of which reduce the dislocation density. In contrast, the increase in hardness outside the SZ arises from the presence of numerous subgrains (high dislocation density) and retained or reprecipitated second-phase particles (Gupta et al. 2025b; Tiwari et al. 2020). Although grain refinement does occur in the SZ, the hardness does not increase as expected from Hall–Petch strengthening because the loss of precipitate- and solute-strengthening contributions dominates over the Hall–Petch effect. In previous studies on FSPed AA6061 composites (with and without graphene), SZ hardness decreased to 84–103 HV compared with ~ 128 Hv for the as-received AA6061-T6 (Pouraliakbar et al. 2024). Similar reductions (50–90 HV) have been widely reported due to dissolution of nanoscale β″ strengthening precipitates during FSP (Tiwari et al. 2020). The high initial hardness of AA6061-T6 originates from β″ precipitates formed during T6 aging. Additional discussion of the hardening and softening mechanisms is provided in the next section. Overall, Fig. 5 establishes the baseline microstructure and particle distribution before CT-FSP, which critically influences second phase particles dissolution, reprecipitation behavior, and the resulting mechanical softening during processing.

### Second phase particles and DRX behavior of FSPed sample

**Fig. 6:**
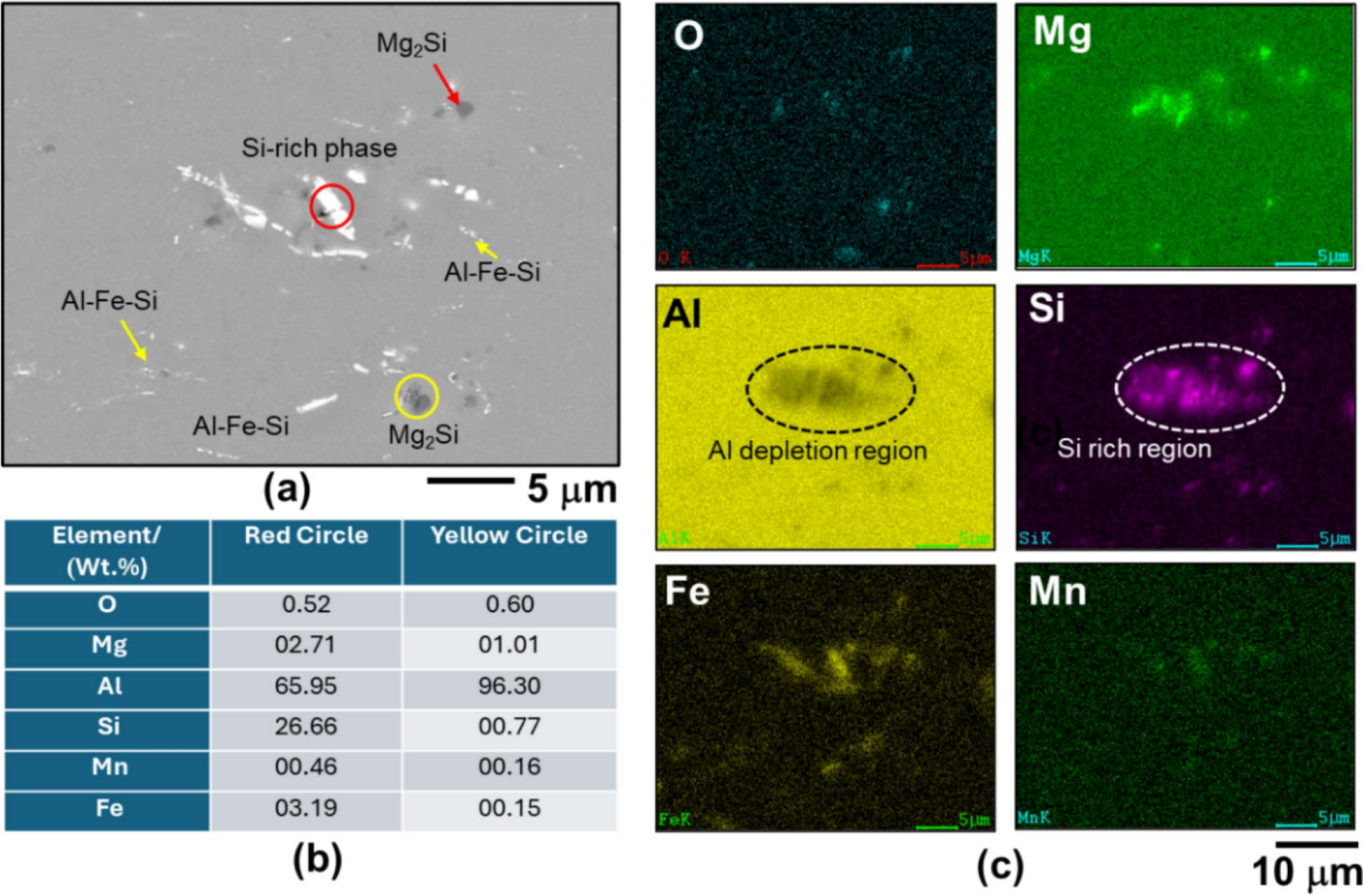
Evolution of precipitates/intermetallics in the CT-FSPed sample: **a**, **b** SEM micrographs and EDS point analyses showing the elemental signatures of grey (Si-rich/Al–Fe–Si) and dark (Mg_2_Si) contrast particles. **c** EDS area mapping illustrating the elemental distribution corresponding to the grey and dark contrast features

Figure 6 presents SEM and EDS analyses highlighting the morphological and compositional evolution of intermetallic particles after CT-FSP at 600 RPM. Figure 6a shows a representative micrograph from the bottom region (TMAZ). Although SEM images were obtained from multiple locations (surface, center, RS, and AS), the bottom region is shown here because it provides the clearest visualization of the second phase particles features. As noted earlier in Fig. 5a-b, dark-contrast particles correspond to Mg_2_Si, while grey-contrast particles correspond to Al–Fe–Si intermetallics. Similar contrast behavior is observed after CT-FSP. In Fig. 6a, the coarser particle marked with a red circle is identified as a Si-rich phase. Dark and grey particles are also highlighted, corresponding to Mg_2_Si and Al–Fe–Si, respectively.

Figure 6b shows the elemental compositions of the red- and yellow-circled second-phase particles. The yellow-circled particle (dark contrast) exhibits higher Mg and Si contents compared with Fe and Mn, consistent with Mg_2_Si. In contrast, the red-circled particle (grey contrast) is coarser and shows a significantly elevated Si content (~ 26%), suggesting the presence of an Al–Si intermetallic phase, which is relatively uncommon. Similar Si-rich coarse particles were previously reported in RT-FSPed AA6xxx (Gupta et al. 2025b). In the as-received sample, Mg_2_Si particles exhibit Mg contents ranging from 1.06 to 3.73 wt.% and Si contents from 2.22 to 6.28 wt.%. After CT-FSP, the measured Mg (~ 1.01 wt.%) and Si (~ 0.77 wt.%) levels appear more uniformly distributed within the aluminum matrix. Figure 6c presents the elemental distribution corresponding to the SEM image. The presence of oxygen correlates with the Mg_2_Si (dark-contrast) precipitates, and magnesium content is notably higher in these regions. Interestingly, the central area of the SEM image exhibits reduced Al signal and elevated Si content, consistent with the Si-rich regions identified in the mapping. The Fe distribution aligns well with the grey-contrast particles, confirming their identity as Al–Fe–Si intermetallics. In several locations, an increased Mn concentration is also observed, further supporting the classification of these grey-contrast features. Overall, the CT-FSPed sample contains both Al–Fe–Si and Mg_2_Si particles, consistent with the microstructural observations made in the prior sections.

**Fig. 7:**
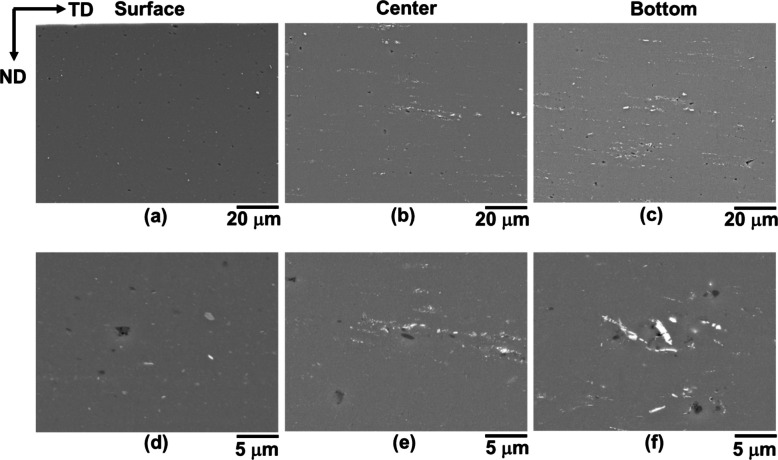
Through-thickness second phase particle distribution: SEM images of the **a** surface, **b** center, and **c** bottom regions. **d**-**f** high magnification images of the surface, center and bottom regions, respectively. Differences in particle behavior are evident across the thickness; the SZ exhibits refined precipitates, whereas the TMAZ contains relatively coarser second-phase particles. Dark-contrast particles correspond to Mg_2_Si, while grey-contrast particles correspond to Al–Fe–Si intermetallics

Figure 7 illustrates the through-thickness distribution of second phase particles in the CT-FSPed sample. The SEM images obtained from the surface, center, and bottom regions show a distinct spatial gradient in particles morphology and spacing. The surface and center exhibit a finer and more uniformly dispersed population of second-phase particles both grey Al–Fe–Si and dark Mg_2_Si consistent with enhanced fragmentation and reprecipitation under cryogenic conditions (Fig. 7a − b). In contrast, the bottom region (TMAZ/near base material) retains larger and more sparsely distributed coarse particles (Fig. 7c). These observations agree with previously reported findings at 800 RPM, where CT-FSP promoted particle fragmentation, reduced equivalent circle diameters (ECD), and improved particles uniformity in the SZ. However, at the lower tool speed of 600 RPM, the through-thickness contrast becomes more pronounced, reflecting differences in strain intensity and cooling efficiency. In the previously reported 800-RPM CT-FSP study (Gupta et al. 2025a), the nearest-neighbor distances for both Al–Fe–Si and Mg_2_Si particles decreased significantly compared with the as-received sample. For Al–Fe–Si, the spacing decreased from 5.83 ± 5.11 μm (initial) to 4.46 ± 3.59 μm (surface), 3.54 ± 3.15 μm (center), and 3.56 ± 2.50 μm (bottom). For Mg_2_Si, spacing decreased from 7.30 ± 7.77 μm to 4.80 ± 3.49 μm (surface), 4.94 ± 3.82 μm (center), and 4.14 ± 4.39 μm (bottom). Particle sizes, expressed as equivalent circle diameters (ECD), were also reduced after CT-FSP, with both Al–Fe–Si and Mg_2_Si refined to ~ 0.50–0.60 μm (Gupta et al. 2025a). In the present study, similar image-segmentation techniques were used to calculate ECD and nearest-neighbor distances, as described in Sect. “Image segmentation for second phase particles analysis”. Overall, Fig. 7 confirms the same dominant mechanisms previously reported particles fragmentation, redistribution, and localized dissolution/reprecipitation while demonstrating that reducing the rotation speed to 600 RPM increases the through-thickness heterogeneity in particle size and spacing.

**Fig. 8:**
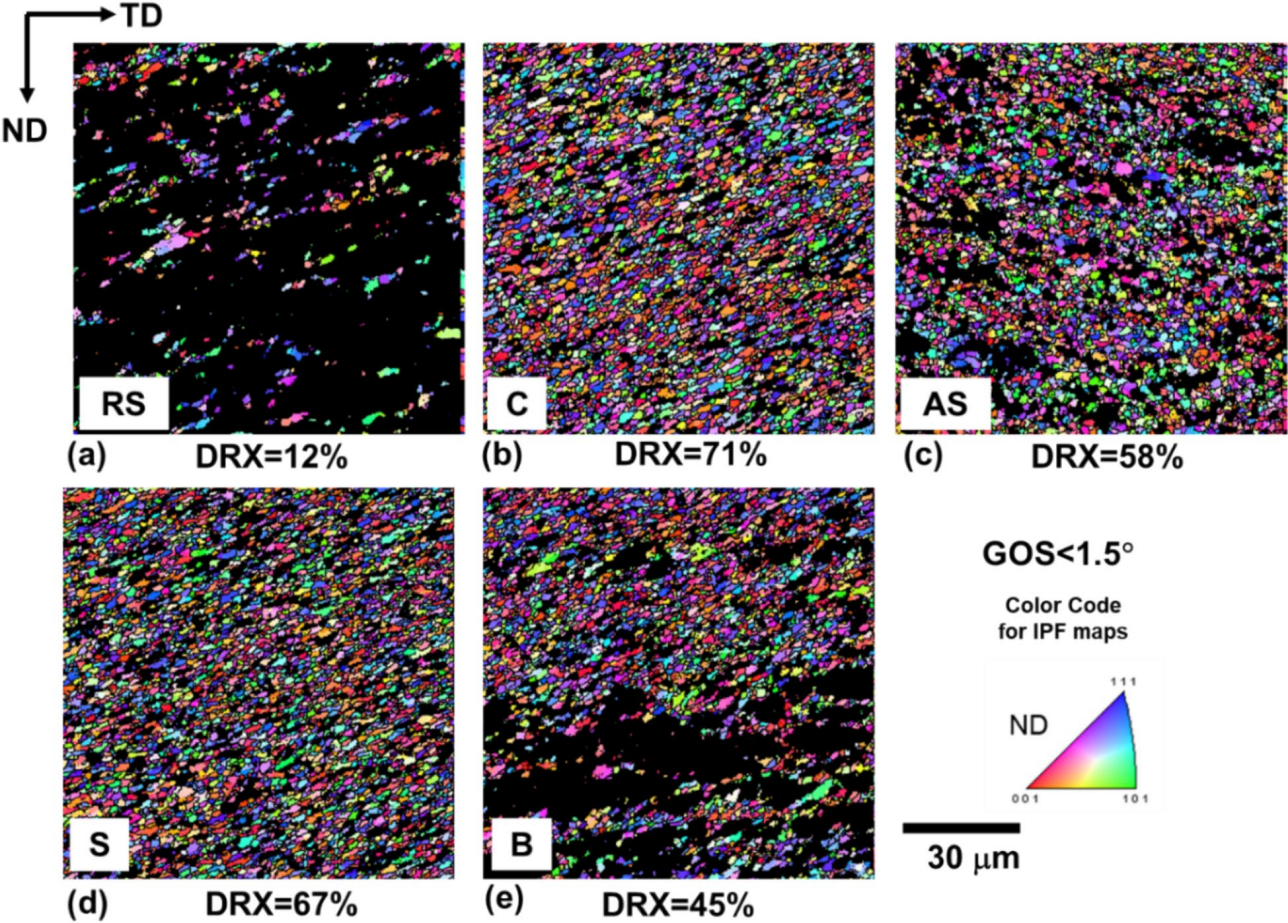
IPF maps of recrystallized grains observed at the **a** RS, **b** center, **c** AS, **d** surface, and **e** bottom regions. A GOS ≤ 1.5° criterion was used to distinguish recrystallized grains from the deformed matrix. The DRX fractions for each region are listed below the respective subfigures. Higher DRX fractions are observed in the SZ, whereas the TMAZ shows substantially lower DRX

The IPF maps segmented using the GOS ≤ 1.5° threshold (Fig. 8) quantify the recrystallized (DRX) grain fractions across the RS, center, AS, surface, and bottom regions. High DRX fractions are observed at the center and surface positions (67–71%), while the RS, AS, and bottom regions show lower DRX fractions (12%, 58%, and 45%, respectively), indicating retained deformation substructure in these areas. This distribution is consistent with prior 800-RPM CT-FSP results, where extensive DRX in the SZ was linked to notable grain refinement and SZ softening. However, the present 600-RPM data exhibit greater positional variability in DRX fraction, including reduced DRX in certain lateral (Fig. 8a, c) and bottom (Fig. 8e) locations. Mechanistically, these differences reflect the reduced frictional heating at lower tool rotation speeds, which shifts the competition between DRX, DRV, and subgrain formation. Regions with high DRX correspond to ultrafine equiaxed grains and more advanced particle fragmentation/refinement, contributing to improved strain distribution and formability. Conversely, regions with lower DRX retain strong orientation gradients, subgrains, and coarser second phase particles, which increase local hardness and may serve as crack-initiation sites during forming.

Yadav et al. (2018) studied the DRX behavior in the FSPed Al-Zn samples in which the dominant DRX mechanism was CDRX, assisted by DRV. High stacking fault energy of aluminium promotes dislocation rearrangement into subgrain boundaries by DRV, which progressively increases in misorientation. These subgrains gradually transform into HAGBs, leading to fine equiaxed grains during FSP (Yadav et al. 2018). In another investigation, it was concluded that CDRX was dominant based on EBSD and TEM evidence for the AA6063-tungustun reinforced FSPed sample (Ali et al. 2021). Electron microscopy showed progressive subgrain formation and rotation rather than nucleation of new grains. It was observed that a high fraction of LAGBs transforming into HAGBs, consistent with dislocation rearrangement by DRV. The absence of bulged grain boundaries or necklace structures ruled out DDRX, supporting CDRX as the primary mechanism (Yadav et al. 2018). Based on the previous reports, it can be concluded that the CDRX is dominant softening mechanism in the present investigation. Together, Figs. 7 and 8 demonstrate the coupled interplay between second phase particles evolution and DRX behavior at 600 RPM, offering a clear explanation for the subtle but logical differences in hardness maps and microstructural trends relative to the previously reported 800-RPM CT-FSP results (Gupta et al. 2025a).

### Image segmentation for second phase particles analysis

A deep-learning (DL)-based image segmentation framework was developed to extract and quantify second-phase particles from SEM micrographs. All stages of the pipeline including dataset construction, model training, and inference were implemented in Python using TensorFlow and the segmentation-models library, with custom preprocessing and post-processing routines tailored to metallographic contrast conditions. SEM images were first resized to 1024 × 1024 pixels and then partitioned into 256 × 256 patches with 50% overlap to ensure consistent representation of fine precipitates and to substantially increase the effective training set size. Ground-truth masks were generated using the multi-Otsu thresholding approach combined with the preprocessing strategy described in Thool et al. (2025). Each segmentation mask was label-encoded and reshaped into a three-channel categorical tensor to enable multiclass semantic segmentation. A U-Net architecture was employed for pixel-wise segmentation. The model comprises a symmetric encoder–decoder structure, the encoder extracts multiscale features via convolution and pooling operations, while the decoder progressively restores spatial resolution using upsampling layers with skip connections to retain boundary and morphological detail. Input patches were normalized and interpreted via a softmax output layer corresponding to three semantic classes: background, Al–Fe–Si, and Mg_2_Si. A hybrid loss function combining Dice loss with categorical focal loss was used to mitigate class imbalance and improve boundary definition for small or low-contrast precipitates. Additional details regarding the segmentation methodology can be found in Thool et al. (2025). Each segmented image was subsequently analyzed using the scikit-image library (Walt et al. 2014) to extract particle morphology metrics. For every labeled precipitate, the equivalent circular diameter (ECD) and nearest-neighbor spacing were calculated. These quantitative results, compiled across all sampled regions, yielded particle statistics for the surface (SZ), center (SZ), and bottom (TMAZ), as summarized in Table 1.

**Table 1:** Nearest-neighbor spacing and equivalent circular diameter (ECD) of second-phase particles for the as-received and 600-RPM CT-FSPed samples

All units in μm	Initial Sample	CT-FSP Sample		
		**Surface**	**Center**	**Bottom**
Nearest distance (Al–Fe-Si)	5.83 ± 5.11	4.43 ± 2.75	5.18 ± 4.40	3.73 ± 2.63
Nearest distance (Mg_2_Si)	7.30 ± 7.77	8.02 ± 5.62	11.38 ± 8.87	7.79 ± 7.34
ECD (size) Al–Fe-Si	0.97 ± 0.63	0.63 ± 0.40	0.77 ± 0.50	0.70 ± 0.43
ECD (size) Mg_2_Si	0.8 ± 0.47	0.65 ± 0.35	0.77 ± 0.45	0.61 ± 0.31

**Fig. 9:**
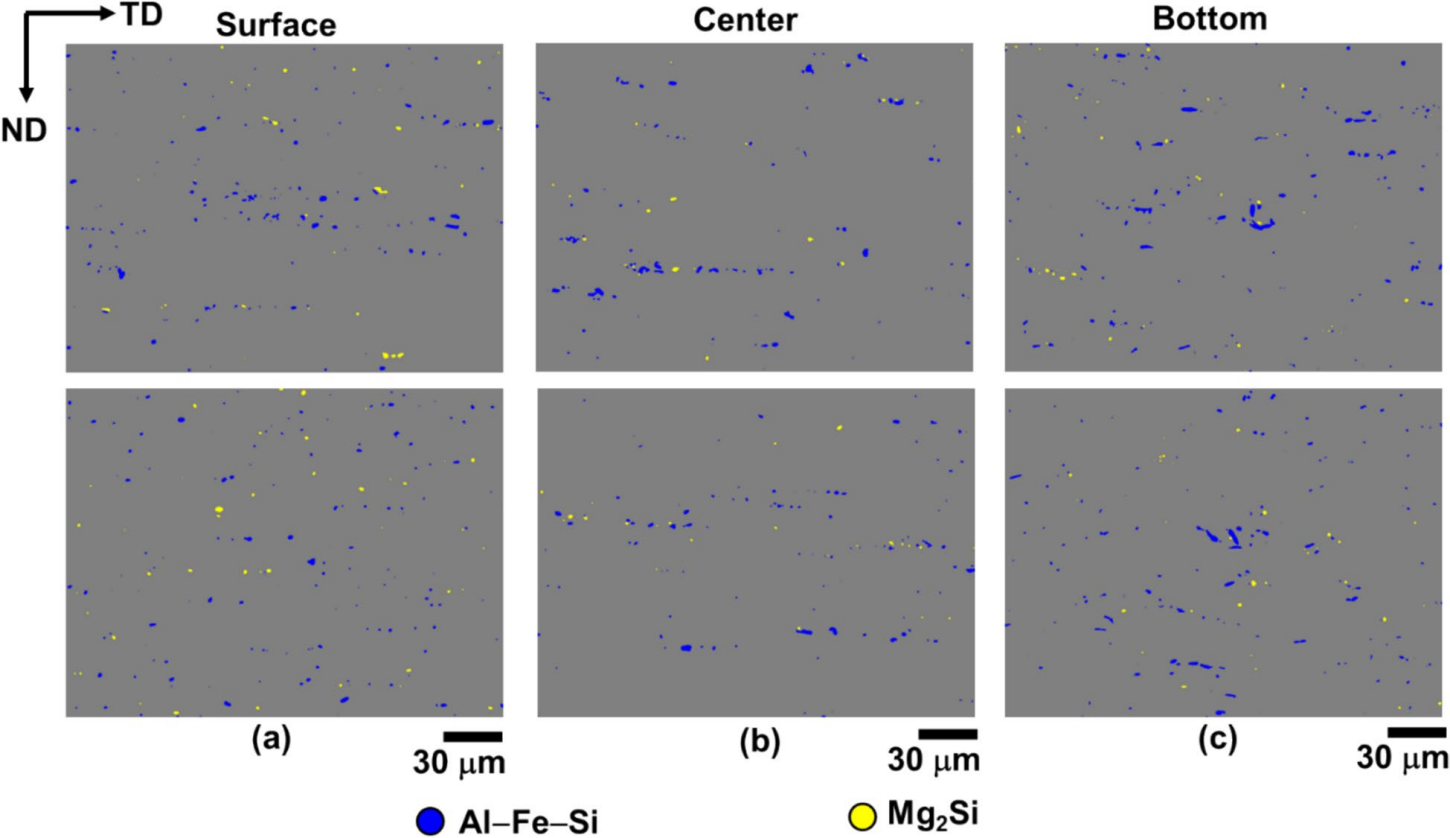
Development of second-phase particles/precipitates in the CT-FSPed sample: **a** surface region (SZ), **b** center region (SZ), and **c** bottom region (TMAZ). Note: The images shown here are DL-segmented representations of SEM micrographs acquired at 1000 × magnification. Blue particles correspond to Al–Fe–Si intermetallics, while yellow particles represent the Mg_2_Si phase

Figure 9a–c show the DL-segmented precipitate maps for the surface (SZ), center (SZ), and bottom (TMAZ) regions, respectively. The surface region (Fig. 9a) contains predominantly fine, uniformly dispersed particles with a mixture of equiaxed and slightly elongated morphologies. The center region exhibits a non-uniform distribution of second-phase particles, characterized by irregular boundaries and non-equiaxed morphologies (Fig. 9b). In contrast, the bottom region (TMAZ, Fig. 9c) retains a higher fraction of coarse and sparsely distributed particles, consistent with reduced fragmentation and limited dissolution in that region. As indicated by the IPF map, the bottom corresponds to the TMAZ, where both fine and coarse grains coexist. However, dissolution and fragmentation of second-phase particles are less pronounced here, resulting in the persistence of coarse intermetallics. In the as-received material, the mean nearest-neighbor distances were approximately 5.83 ± 5.11 µm for Al–Fe–Si and 7.30 ± 7.77 µm for Mg_2_Si, with corresponding mean ECDs of 0.97 ± 0.63 µm and 0.80 ± 0.47 µm, respectively (Gupta et al. 2025a). After 600-RPM CT-FSP, both particle types became finer and more closely spaced in the SZ (surface and center) relative to the initial condition. For Al–Fe–Si, the nearest-neighbor spacing decreased at surface, center and bottom, with the smallest spacing observed at the bottom (≈3.73 ± 2.63 µm). Mg_2_Si nearest-neighbor distances showed local variations but generally increased in the processed regions compared with the initial material. This trend suggests substantial dissolution of Mg_2_Si during FSP, with limited reprecipitation due to rapid cryogenic cooling.

Particle sizes for both Al–Fe–Si and Mg_2_Si decreased markedly after CT-FSP, with the smallest mean ECDs observed at the surface (Al–Fe–Si: ≈0.63 ± 0.40 µm) and bottom (Mg_2_Si: ≈0.65 ± 0.35 µm) regions, respectively. The center and bottom regions for Al–Fe–Si exhibited slightly larger ECDs than the surface but remained significantly smaller than those in the as-received material. These quantitative results indicate that CT-FSP at 600 RPM promotes fragmentation and partial dissolution of coarse intermetallics within the SZ, producing a refined precipitate population. Fragmentation is strongest at the surface where direct tool contact generates higher strain and least effective at the bottom/TMAZ, where larger particles persist. The spatial variations in particle size and spacing correlate closely with DRX fraction and local strain/thermal gradients, as shown in Figs. 2, 3, 4, 5, 6, 7 and 8.

### Effect of second phase particles on microhardness

**Fig. 10:**
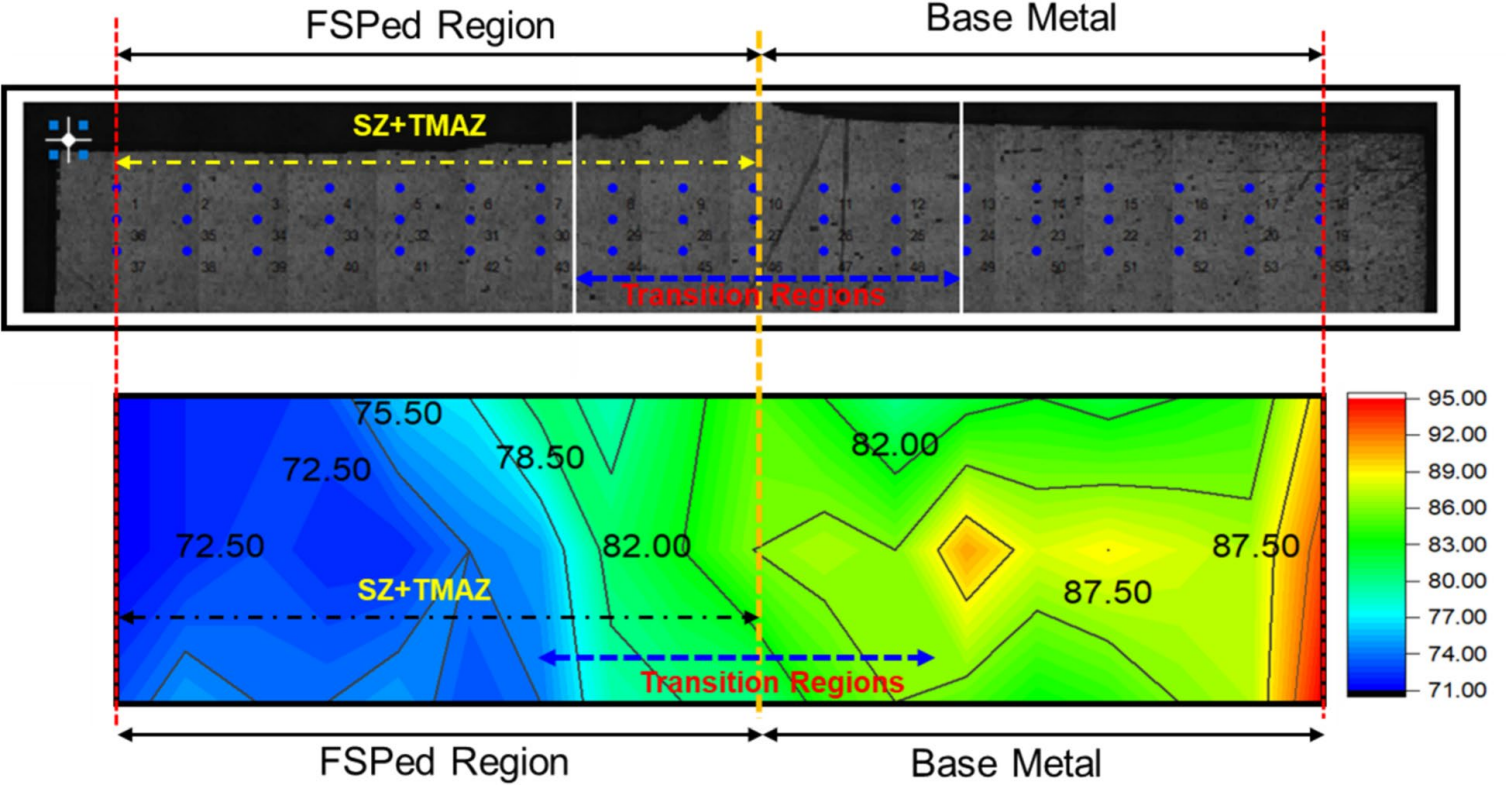
Optical micrograph and hardness contour showing the microhardness transitions from SZ-TMAZ-BM regions. Blue color dashed arrows show the microhardness transition from TMAZ to BM region. The numerical values written inside the hardness contours are microhardness values at respective positions in Hv. Yellow color dashed arrow in optical micrographs shows the SZ + TMAZ regions

The relationship between microhardness, the FSPed microstructure, and second-phase particles is discussed in this section. The mechanical properties of AA6xxx alloys are primarily governed by solid-solution strengthening, Hall–Petch strengthening, precipitation hardening, and dislocation hardening (Davidkov et al. 2012). In the present study, Hall–Petch and precipitation strengthening are identified as the dominant mechanisms. However, in the SZ, the loss of precipitate/solute strengthening outweighs the gains expected from Hall–Petch strengthening. Because the as-received AA6xxx sheet was in the solution-annealed state, artificial aging may have occurred in the TMAZ due to thermal exposure during FSP, contributing to the observed increase in hardness. Accordingly, an additional microhardness measurement was performed to examine the hardness transition across the SZ–TMAZ–BM regions. As shown in Fig. 10, two distinct regions*—“FSPed region” and “BM region”*—are visible in the optical micrograph. The figure presents the optical micrograph together with the corresponding microhardness contour map for the CT-FSPed AA6xxx sample. Only half of the FSPed zone (5 mm) was evaluated along with the adjacent BM region. The blue dashed arrows in Fig. 10 indicate the hardness transition from TMAZ to BM, showing a progressive increase from low to high hardness values. In the BM, hardness contour colors range from green to yellow, whereas in the SZ + TMAZ regions they range from dark blue to light blue, indicating relatively lower hardness in comparison. The BM exhibited homogeneous hardness values, consistent with previously reported values of 87 ± 6 Hv (Gupta et al. 2025b). Regions exhibiting higher DRX fractions, finer grain sizes, and increased interparticle spacing of Mg_2_Si (8.02–11.38 μm) showed reduced hardness values (surface and center). The increased spacing and decreased Mg_2_Si particle size (0.65–0.77 μm) indicate partial dissolution as well as reprecipitation. Within the SZ, the Al–Fe–Si particles also showed reduced interparticle spacing (4.43–5.18 μm) and smaller equivalent circle diameters (0.63–0.77 μm), consistent with particle fragmentation. However, despite their fragmentation, these particles do not significantly contribute to strengthening in the SZ (surface and center regions).

In the other hand, regions with reduced DRX (RS/AS and bottom) retain coarser particles cause the evolution of the dislocation structures. Although the particles characteristics were not discussed for RS and AS regions, the observable features of second phase particles for the bottom region resemble the TMAZ (Figs. 7c and 9c). Grain boundary pinning by reinforcement particles or coarse second phase particles inhibit dislocation motion. Hence, an increase in dislocation density due to thermal expansion mismatch between the matrix and particles, observed and the formation of geometrically necessary dislocations (GNDs) arising from strain incompatibility during deformation (Ali et al. 2021).

## Conclusions

A heterogeneous microstructure was obtained after CT-FSP of the AA6xxx sample. Orientation microscopy, SEM/EDS, and DL-based image segmentation were used to quantify grain characteristics, DRX behavior, and precipitate evolution, respectively. The key findings are summarized as follows:

1. Cryogenic FSP at 600 RPM (500 mm·min^-1^, LN_2_ cooling) produces a finely equiaxed stir zone. The surface and center regions exhibit average grain sizes of ≈1.6–1.8 µm, whereas the bottom/TMAZ retains coarser grains (~ 4.4 µm). The reduced rotational speed lowers heat input relative to higher-speed CT-FSP, thereby stabilizing the refined microstructure.
2. DRX is the primary restoration mechanism in the SZ under 600-RPM CT-FSP, confirmed by high HAGB fractions, low KAM values at the center, and large DRX fractions (GOS ≤ 1.5°) at surface and center locations. Spatial variations in DRX particularly reduced DRX at RS, AS, and bottom positions reflect local competition between DRX, CDRX/DRV, and subgrain formation dictated by heterogeneous strain fields and cryogenic cooling gradients.
3. Second-phase particles (Al–Fe–Si and Mg_2_Si) undergo significant fragmentation, partial dissolution, and redistribution during 600-RPM CT-FSP. This results in finer, more uniformly dispersed precipitates in the SZ, while the bottom/TMAZ retains larger particles. In the as-received material, mean ECDs were 0.97 ± 0.63 µm (Al–Fe–Si) and 0.80 ± 0.47 µm (Mg_2_Si), with nearest-neighbor distances of 5.83 ± 5.11 µm and 7.30 ± 7.77 µm, respectively. After CT-FSP, both ECDs and nearest-neighbor distances decreased across the surface, center, and bottom, indicating strong fragmentation and partial dissolution most pronounced at the surface and decreasing toward the bottom/TMAZ.
4. The combined effects of particle refinement, dissolution, and DRX lead to a spatially heterogeneous hardness profile. SZ softening (lower Hv) is attributed to second phase particles dissolution and DRX-induced softening, whereas TMAZ hardening arises from retained coarse particles, subgrains, and higher dislocation densities. The quantitative second phase particles metrics provide insight into the observed hardness gradients and offer guidance for optimizing process parameters to balance grain refinement and precipitation strengthening in automotive sheet applications.

## Data Availability

Data can be made available on reasonable requests.
